# Rhythmic visual stimulation enhances visual search via occipito-parietal alpha modulation: an electroencephalographic study

**DOI:** 10.3389/fnins.2026.1780980

**Published:** 2026-04-15

**Authors:** Hongwei Wang, Suya Bao, Zihan Gang, Bo Gao, Wenliang Fu, Yuhao Chi, Mingzhe Zhang, Yue Wu, Haowei Wu, Huan Niu, Chao Zhang, Donggang Xu, Yongcong Shao, Weiwei Xing

**Affiliations:** 1 Beijing Institute of Basic Medical Sciences, Beijing, China; 2 Department of Critical Care Medicine, The Second Affiliated Hospital of Chongqing Medical University, Chongqing, China; 3 School of Psychology, Beijing Sport University, Beijing, China; 4 School of Software, North University of China, Taiyuan, Shanxi, China

**Keywords:** alpha oscillations, EEG, rhythmic stimulation, visual flicker entrainment, visual search

## Abstract

**Introduction:**

As a non-invasive neuromodulation technique, visual flicker entrainment has demonstrated considerable potential in enhancing basic visual perception; however, the neurophysiological mechanisms underlying these effects remain unclear. This study investigated whether rhythmic visual stimulation at individualized alpha frequencies can improve low-contrast visual search performance by selectively modulating alpha-band neural oscillations.

**Methods:**

Forty-three healthy male participants completed a low-contrast visual search task under two conditions: personalized rhythmic flicker and arrhythmic (random) flicker. Behavioral performance was evaluated using reaction time, accuracy, and perceptual sensitivity. Simultaneously, high-density electroencephalographic data were recorded. Neural activity was quantified using power spectral density analysis across delta, theta, and beta frequency bands. Neural oscillatory characteristics were compared across prefrontal, central, parietal, and temporal areas under different flicker conditions.

**Results:**

Behaviorally, performance under the rhythmic flicker condition was significantly enhanced relative to that under the random flicker condition, as reflected by significantly reduced reaction times. Electrophysiologically, rhythmic flicker elicited a significant increase in overall alpha power (*F*_(1, 42)_ = 6.90, *p* = 0.012, η^2^_p_ = 0.14). Critically, this effect was region-specific: a significant Condition × Region interaction (*F*_(3, 126)_ = 7.83, *p* < 0.001). Alpha power in the occipital region was significantly higher during rhythmic flicker compared to arrhythmic flicker (mean difference = 0.51 μV^2^, *p* = 0.007, uncorrected). The analysis of the parietal region using the Wilcoxon signed-rank test revealed a significant moderate increase (*Z* = 2.15, *p* = 0.031, uncorrected). No significant differences between conditions were found in the frontal or temporal regions (all *p s* > 0.05). Additionally, a significant Region × Electrode interaction was observed (*F*_(6, 252)_ = 12.83, *p* < 0.001, η^2^_p_ = 0.21). This indicates that the distribution of alpha power across electrodes differed by brain region. Furthermore, enhanced parietal alpha power was significantly correlated with a reduced reaction time (Pearson’s *r* = −0.35, *p* = 0.021). By contrast, no significant modulation by rhythmic stimulation was observed in delta, theta, or beta bands (all Condition main effects and Condition × Region interactions, *p* > 0.05).

**Conclusion:**

Individualized alpha-frequency visual flicker entrainment effectively enhances performance in male participants on complex visual search tasks, with the behavioral benefits mediated by selective modulation of neural oscillations in the parietal alpha band. These findings provide mechanistic electrophysiological evidence that rhythmic stimulation improves visual cognition by modulating frequency- and region-specific neural dynamics.

## 1 Introduction

Visual search constitutes a core component of visual cognition, requiring the seamless integration of perceptual processing, memory retrieval, and executive control to identify targets or extract task-relevant information from complex visual scenes (Treisman and Gelade, 1980; Wolfe et al., 1989; Wolfe and Horowitz, 2017). However, in naturalistic environments, search efficiency and accuracy are severely compromised by degraded sensory conditions—such as variable illumination, fog, and low-light conditions—that substantially reduce target-background contrast. Although classical frameworks, including feature integration theory, have provided important insights into visual search under high-salience conditions, the cognitive and neural mechanisms governing visual search under low-contrast constraints remain poorly understood. However, existing research has predominantly focused on simple detection tasks performed under ideal viewing conditions, leaving a critical gap in our understanding of how reduced contrast impacts complex search processes that require concurrent memory retrieval, attentional control, and decision-making (Wolfe, 2021; Eckstein, 2011). Therefore, systematic investigation of the neurocognitive mechanisms underlying low-contrast visual search—and the development of targeted neuromodulatory interventions, such as those leveraging neural oscillations (e.g., alpha rhythms)—are essential for advancing theoretical models under ecologically valid conditions and establishing evidence-based strategies to enhance operational performance in real-world scenarios.

Neural oscillations in the alpha frequency range (8–13 Hz) play a central role in regulating visual perception through phase-dependent inhibitory mechanisms (Hanslmayr et al., 2005; Sokoliuk and VanRullen, 2016; Chota and VanRullen, 2019; Samaha et al., 2015). When driven by external stimuli such as visual flickers, alpha oscillations can selectively suppress the processing of irrelevant or distracting information. For example, studies employing the Eriksen flanker task have demonstrated that visual entrainment at the alpha frequency selectively inhibits the perception of distractors, whereas stimulation at higher frequencies fails to produce this effect (Wiesman and Wilson, 2019). Furthermore, entrainment-induced responses in the primary visual cortex have been shown to correlate significantly with behavioral congruency effects, providing causal evidence for the role of alpha oscillations in attentional filtering and distractor suppression (Wiesman and Wilson, 2019; Helfrich et al., 2014).

Despite robust evidence linking alpha oscillatory dynamics to spatial attentional (Peylo et al., 2021; Busch et al., 2009), the effectiveness of alpha entrainment in enhancing visual discrimination remains debated (de Graaf and Duecker, 2022). Nevertheless, findings from binocular rivalry tasks indicate that alpha-frequency entrainment reliably accelerates perceptual alternations, supporting the notion that alpha oscillations regulate visual awareness through the periodic modulation of information accumulation (Torralba Cuello et al., 2022).

These findings suggest that alpha oscillations exert a regulatory influence on visual perception, but their functional contribution under demanding perceptual conditions—such as low-contrast visual search—remains to be fully elucidated. Visual flicker entrainment is a non-invasive neuromodulation technique that drives endogenous alpha oscillations through rhythmic light stimulation, thereby offering a powerful causal tool for investigating its role in perceptual regulation (Herrmann, 2001; Notbohm et al., 2016; Mathewson et al., 2011). From a neurodynamic perspective, the efficacy of neural entrainment follows the principle of “*Arnold tongue*” (Thut et al., 2011), whereby optimal neural entrainment occurs when the external stimulation frequency aligns with the individual’s intrinsic alpha frequency (typically 8–13 H) (Thut et al., 2011). This principle has been demonstrated across different neuromodulation modalities, including transcranial magnetic stimulation (Trajkovic et al., 2024) and visual flicker (Notbohm et al., 2016). Under such frequency-matched conditions, even moderate-intensity stimulation can induce stable phase locking of neural oscillations (Notbohm et al., 2016), potentially implementing a pulsed-inhibition mechanism that regulates cortical information flow (Mathewson et al., 2011).

Critically, effective entrainment depends on accommodating individual neurophysiological traits. The individualized alpha frequency (IAF) exhibits substantial and stable inter-individual differences (Grandy et al., 2013b), which are closely associated with visual temporal resolution (Deodato and Melcher, 2024). Furthermore, the pre-stimulus alpha phase has been shown to predict visual awareness outcomes (Mathewson et al., 2009). These findings underscore the importance of individualized rhythm matching for optimizing entrainment efficacy and suggest that susceptibility to neuromodulation can be predicted from baseline electroencephalographic (EEG) patterns (Trajkovic et al., 2024).

Given the decisive role of the IAF matching in efficient neural entrainment, this study adopted the “entrainment-probe” paradigm to examine whether individualized alpha-frequency visual entrainment can enhance performance in low-contrast visual search tasks and to elucidate the underlying neural mechanisms. Specifically, we address two core research questions: (1) Can individualized alpha entrainment effectively improve visual search performance under low-contrast conditions, as reflected by enhanced perceptual sensitivity (d’)? (2) Is the underlying neural mechanism characterized by the coordinated enhancement of alpha oscillatory activity within the sensorimotor coordination network, particularly involving the occipital and parietal regions? By integrating neural entrainment techniques with alpha oscillation mechanisms, this study seeks to provide novel evidence for understanding how rhythmic stimulation optimizes complex visual cognition through the targeted modulation of specific brain network dynamics.

## 2 Materials and methods

### 2.1 Participants

Sample size estimation was performed using G*Power 3.1.9.7 based on a matched-pairs *t*-test (two-tailed), with an assumed effect size d_z_ of 0.5, significance level *α* of 0.05, and statistical power of 0.8. The analysis indicated that a minimum of 34 paired participants was required to detect the anticipated effects.

In our study, a total of 43 healthy male participants were recruited (mean age ± standard deviation [SD]: 24.16 ± 1.25 years). All participants met the following screening criteria: (1) age >18 years; (2) no history of cardiovascular, hepatic, endocrine, neurological, or psychiatric conditions that could influence study outcomes; (3) no current use of prescribed medication; (4) no history of alcohol or substance abuse or dependence; and (5) no personal or familial history of photosensitive epilepsy.

### 2.2 Experimental task

**Figure 1:**
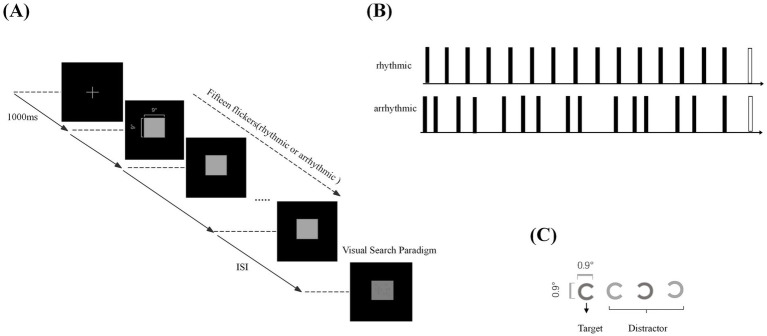
Experimental paradigm flowchart: **(A)** displays the flow of the experimental paradigm. **(B)** Illustrates the different temporal patterns of flicker stimulation. **(C)** Shows the target and distractor stimuli used in the visual search task.

The experiment was conducted in a quiet, dimly lit room. The participants were seated at a viewing distance of 60 cm from the display screen and instructed to maintain central fixation throughout each trial. Each trial commenced with a 1.0-s fixation period during which a central gray cross was presented on a black background and blinking was permitted. The fixation cross was then removed, and this was followed by a 1.5-s visual flicker sequence comprising 15 brief presentations of a gray square positioned at the screen center. During the flicker sequence, participants were instructed to fixate on the centrally-presented flickering stimulus and to minimize blinking. Two flicker conditions were utilized: (i) in the rhythmic flicker condition, flashes were delivered at a constant interval corresponding to the participant’s IAF; (ii) in the arrhythmic flicker condition, inter-flash intervals were sampled randomly from an exponential distribution centered on 1/IAF. In both conditions, each flash lasted 17 ms, and the total number of flashes, along with the onset times of the first and last flashes, were identical (de Graaf and Duecker, 2022; Spaak et al., 2014). The detailed trial sequence is illustrated in Figure 1.

Following the flicker sequence, a 100-ms inter-stimulus interval preceded the presentation of the visual search task at the same screen location. The search array contained 13 Landolt-C-like figures, each subtending 0.9° × 0.9°of visual angle (Müller et al., 2015). On 50% of trials, the target stimulus—a dark gray “C” (RGB: 113, 113, 113) with a right-oriented gap—was present. Distractors included three categories: six dark gray “C” figures with left-oriented gaps, three light gray “C” figures (RGB: 118, 118, 118) with left-oriented gaps and three light gray “C” figures (RGB: 118, 118, 118) with right-oriented gaps (Müller et al., 2015). Participants responded to the presence or absence of the target by pressing a key with their right index or middle finger. Prior to the main experiment, all participants completed practice trials until a minimum accuracy criterion of 60% was achieved. Stimulus delivery and response collection were implemented in Python.

### 2.3 Experimental procedure

The experimental procedure comprised three sequential stages: (1) determination of the IAF, (2) a practice session, and (3) the formal experimental task. First, a 3-min eyes-closed resting-state EEG was recorded. Following standard preprocessing, power spectral analysis was performed on occipital electrodes (PO3, PO4, Oz, O1, and O2) to identify the peak frequency within the 8–13 Hz range, which was as the participant’s IAF. To minimize temporal variability between IAF measurement and its experimental application, we implemented a streamlined protocol: immediately after acquiring resting-state EEG, a Python script calculated each participant’s IAF, and this value was applied without delay to configure the rhythmic flicker frequency. To balance frequency precision with stimulus timing constraints, IAF values were discretized to the nearest 0.5 Hz. Next, participants completed a practice block of the visual search task until stable task performance was achieved (accuracy ≥60%), ensuring task familiarity and minimizing learning effects. Finally, in the formal task, participants completed 540 trials divided into nine blocks, alternating between rhythmic flicker and arrhythmic flicker conditions. Each block consisted of 30 trials of a single condition, followed by a 1-min rest period. Each trial presented a sequence of 15 flashes that differed only in the temporal pattern (fixed vs. exponentially randomized intervals), with all other physical properties maintained identical. The visual search array was displayed 100 ms after the final flash. The study protocol was approved by the Beijing Institute of Basic Medical Science (approval number: AF/SC-08/02.381). All participants provided written informed consent prior to participation.

### 2.4 EEG data acquisition

EEG signals were recorded using a NeuroHUB from Neuracle (Neuracle, Inc.), with a sampling rate of 1,000 Hz. Electrodes were placed according to the International 10–20 system, and electrode impedances were maintained below 10 kΩ throughout data acquisition. Offline data preprocessing was performed using MATLAB (MathWorks, Natick, MA, USA) and the EEGLAB toolbox (Delorme and Makeig, 2004). The preprocessing pipeline included down-sampling to 500 Hz, bandpass at 0.5–75 Hz, and notch filtering at 48–52 Hz to remove line noise. Artifactual data segments were identified and removed, and persistently noisy channels (P7 and P8) were interpolated. Subsequently, independent component analysis (ICA) was applied to identify and remove components associated with ocular and muscular artifacts. Components contributing to less than 10% of the total variance were excluded from further analysis.

### 2.5 EEG and Behavioral data analysis

#### 2.5.1 Behavioral data analysis

To mitigate potential practice and ceiling effects, the first and last 30 trials of each experimental block were excluded from the analysis. Additionally, trials with reaction times shorter than 500 ms or longer than 1,500 ms were removed. Paired-sample *t*-tests were conducted to compare behavioral performance between the rhythmic and arrhythmic flicker conditions across three primary metrics: reaction time, accuracy, and d’. While RT and accuracy serve as conventional performance indices, d’ provides a more refined measure by dissociating perceptual sensitivity from response bias, offering a better assessment of visual discriminative ability (Muggleton et al., 2008; Clouter et al., 2017). d’ was calculated as d’ = Z(hit rate) – Z(false alarm rate). The paired-sample t-test on d’ directly tested the hypothesis that alpha entrainment significantly enhances visual search performance, with the significance level (*α*) set at 0.05. In all figures, error bars represent standard error of the mean (SEM), and significance levels are denoted as follows: *p < 0.05, **p < 0.01.

#### 2.5.2 Estimation of individual alpha peak frequency (IAF)

The complete 180-s artifact-free recording (90,000 samples at 500 Hz after down sampling from 1,000 Hz) was analyzed as a single continuous epoch. Following established practices in IAF estimation, EEG time series from five occipital electrodes (PO3, PO4, Oz, O1, and O2) were averaged in the time domain. To robustly estimate the peak frequency, we applied Gaussian smoothing to the power spectrum before peak detection (Grandy et al., 2013a). To balance frequency precision with stimulus timing constraints, IAF values were discretized to the nearest 0.5 Hz. To verify the accuracy of the IAF estimation, we further parameterized the resting-state EEG data using the FOOOF toolbox (Donoghue et al., 2020) and performed a correlation analysis between the peak frequencies extracted by this method and those estimated in our study. The results showed a high degree of consistency between the two methods (*r* = 0.975, *p* < 0.001), confirming the reliability of our IAF determination approach.

#### 2.5.3 Power spectral density (PSD) data analysis

PSD was computed from the preprocessed EEG data using the Fourier transform. For each experimental trial, EEG data were segmented into epochs spanning from 1 s pre-stimulus (fixation period) to 1.5 s after the onset of the first flash (flicker stimulation period), totaling 2.5 s. Alpha power analysis was performed specifically on this flicker stimulation period, which contains approximately 15 alpha cycles at 10 Hz. According to previous research (Klimesch et al., 2007), this duration is sufficient to provide stable and reliable power estimates while capturing sustained oscillatory activity during the task-relevant time window. Power spectral density was computed using periodogram-based estimation with the following specifications: analysis window of 1.5-s flicker stimulation period. In the current analysis, the periodogram method was applied to estimate the PSD of all 59 channels across the delta (1–4 Hz), theta (4–8 Hz), alpha (8–13 Hz), and beta (13–30 Hz) frequency bands.

To analyze the spatial distribution pattern of alpha wave activity, we divided the electrodes of interest into four brain regions based on the international 10–20 system and relevant neuroscientific research: the frontal lobe (Fp1, Fp2, and Fpz), parietal lobe (P3, P4, and Pz), occipital lobe (O1, O2, and Oz), and temporal lobe (T7, T8, and TP7). For region-specific analysis, we computed the mean alpha power within each of these four ROIs. Additionally, we conducted a whole-brain analysis by computing the mean alpha power across all 59 scalp electrodes for each participant. Statistical comparisons between conditions employed Wilcoxon matched-pairs signed-rank tests for both approaches. Among these, parietal electrodes (e.g., P3/P4) were selected due to their central role in sensory integration and visuospatial attention, whereas occipital electrodes (e.g., O1/O2) were selected due to their close association with primary and advanced visual processing. This division facilitated the investigation of differential changes in alpha oscillations across functional brain regions before and after the intervention. To examine the differences in neural oscillatory power between the conditions, a 2 (condition 1: rhythmic flicker and condition 2: arrhythmic flicker) × 4 (brain region: frontal, temporal, parietal, and occipital) × 3 (electrode: three electrodes per region) repeated-measures analysis of variance (rmANOVA) was conducted on computed PSD values. The three electrode positions in the 2 (Condition) × 4 (Region) × 3 (Electrode) design were designated as: Electrode 1 (left hemisphere: Fp1, P3, O1, T7), Electrode 2 (right hemisphere: Fp2, P4, O2, T8), and Electrode 3 (midline: Fpz, Pz, Oz, TP7). These electrodes were nested within each of the four brain regions, yielding 12 total electrode sites. Mauchly’s test was used to assess the sphericity assumption, and Greenhouse–Geisser correction was applied when this assumption was violated. Additionally, exploratory correlation analyses were performed to investigate the associations between changes in PSD values across different frequency bands and behavioral measures. All statistical tests were conducted with a significance threshold of *α* = 0.05.

## 3 Results

### 3.1 Behavioral results

**Figure 2:**
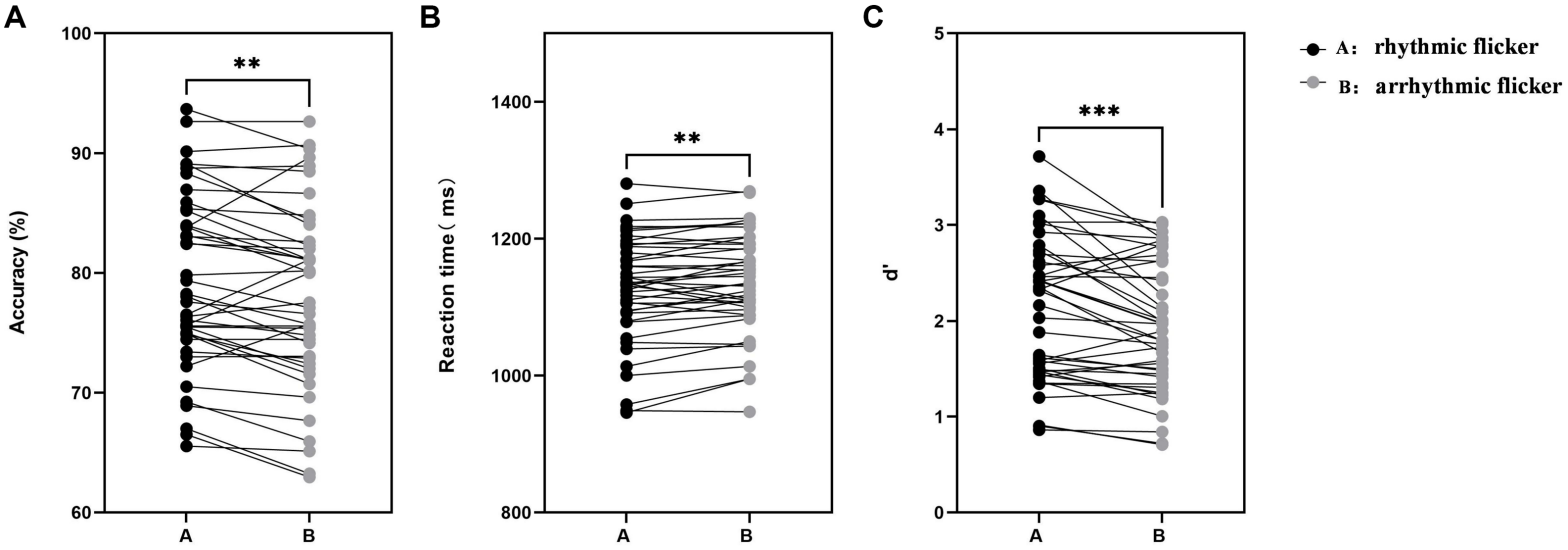
Behavioral performance under rhythmic versus arrhythmic flicker conditions. Each dot represents one participant, with thin lines connecting paired data from the same participant. **(A)** Reaction time (unit: ms) under rhythmic flicker (individualized alpha entrainment) and arrhythmic flicker conditions. Participants demonstrated significantly faster reaction times under the rhythmic flicker condition (*t* (42) = 2.84, *p* = 0.0069). **(B)** Task accuracy (%) for the two conditions. Performance accuracy was significantly higher under the rhythmic flicker condition (*t* (42) = 3.08, *p* = 0.0037). **(C)** Perceptual sensitivity (d’) derived from signal detection theory. Sensitivity was significantly enhanced under the rhythmic flicker condition (*t* (42) = 3.87, *p* < 0.001). Error bars represent the standard error of the mean (SEM). *: *p* < 0.05; **: *p* < 0.01; ***: *p* < 0.001.

To evaluate the impact of individualized alpha entrainment on visual search performance, paired-sample t-tests were conducted comparing the rhythmic flicker and arrhythmic flicker conditions. Behavioral and electrophysiological outcomes are illustrated in Figure 2. The results revealed that, compared with the arrhythmic flicker condition, participants exhibited significantly shorter reaction times (*t*_(42)_ = 2.84, *p* = 0.0069) and significantly higher task accuracy (*t*_(42)_ = 3.08, *p* = 0.0037) under the rhythmic flicker (i.e., alpha entrainment) condition. Importantly, signal detection theory analysis indicated a significant enhancement in participants’ d’ under the rhythmic flicker condition (*t*_(42)_ = 3.87, *p* < 0.001). These findings suggest that individualized alpha-frequency entrainment effectively enhances visual perceptual capacity and improves behavioral performance in visual search tasks.

### 3.2 EEG results

**Figure 3:**
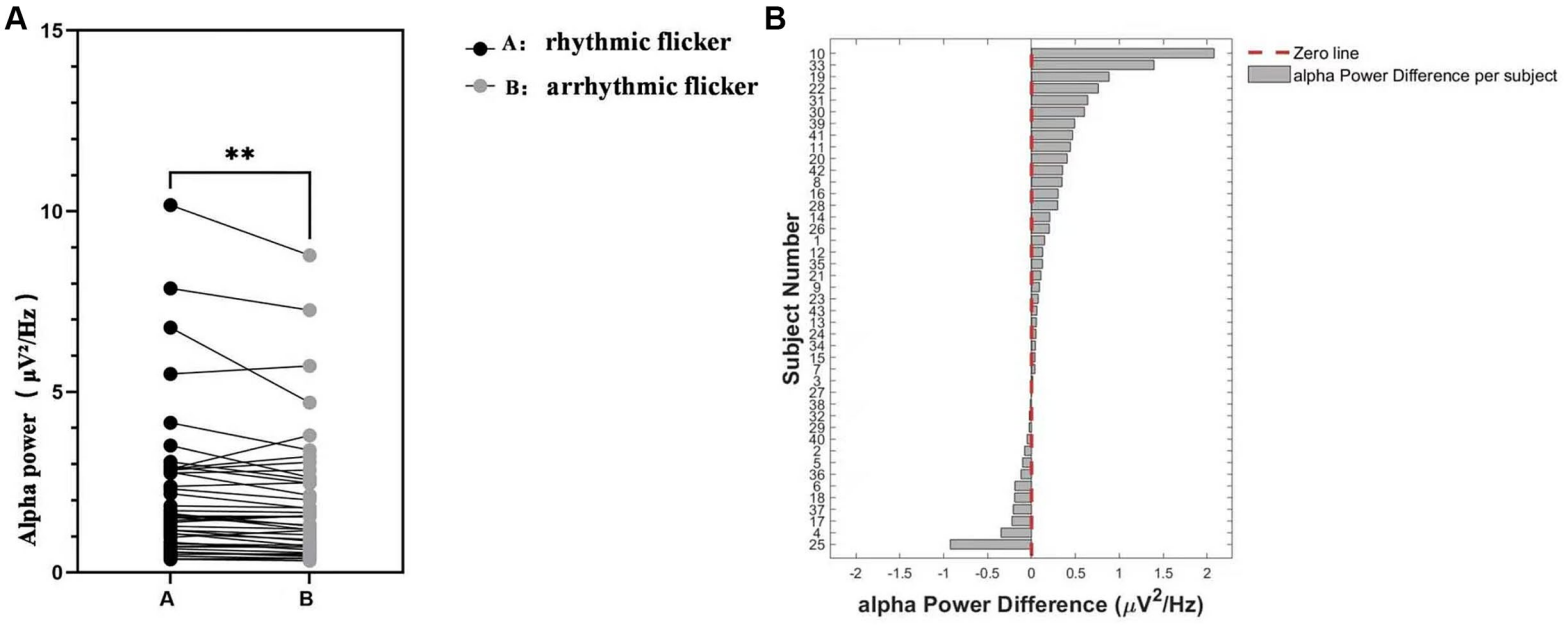
Alpha power modulation by rhythmic visual stimulation at the whole-brain level. **(A)** Comparison of occipital alpha power between rhythmic flicker (black dots) and arrhythmic flicker (gray dots) conditions. Each line represents a single participant. ** indicates significant difference between conditions (*p* < 0.01). **(B)** Distribution of alpha power differences (rhythmic minus arrhythmic) across participants, ranked by effect size. The red dashed line indicates zero difference, and the gray bars represent individual participants. Positive values indicate enhanced alpha power under rhythmic flicker.

To visualize individual-level differences in Alpha power spectral density between rhythmic and non-rhythmic flicker conditions, Figure 3 presents data from a whole-brain channel analysis. Specifically, we first calculated the mean Alpha power spectral density across all 59 scalp electrodes for each participant. As the data violated normality (Shapiro–Wilk test, *p* < 0.0001), we used the Wilcoxon signed-rank test for pairwise comparisons. The results showed that Alpha power spectral density was significantly higher in the rhythmic flicker condition than in the non-rhythmic condition (*Z =* 0.11, *p* = 0.004, *n* = 43). At the individual level, 29 participants (67.4%) showed greater Alpha power spectral density for rhythmic flicker (positive difference), while 13 participants (30.2%) showed the opposite pattern (negative difference). One participant (2.3%) exhibited a near-zero difference. The scatter plot in Figure 3A clearly depicts these individual variations and the correspondence between conditions. Most data points fall above the diagonal line, visually confirming the Alpha enhancement effect under rhythmic flicker. The frequency distribution in Figure 3B further characterizes the distribution of Alpha power spectral density differences. The differences were mainly concentrated in the 0 to 2 μV^2^/Hz range, forming a right-skewed distribution with a peak around 0.5–1.0 μV^2^/Hz. This pattern aligns with the direction of the group-level difference revealed by the Wilcoxon test.

To systematically evaluate the power spectral density characteristics of pre-selected brain regions and specific electrodes under two experimental conditions, Table 1 presents descriptive statistics for each frequency band. This table summarizes the mean and standard deviation (μV^2^/Hz) of power spectral density across four frequency bands and four brain regions (including three electrode positions), providing a data foundation for subsequent hypothesis testing. Region-specific condition effect analyses revealed significant differences between rhythmic and non-rhythmic flicker conditions in the occipital region (*t* (42) = 2.813, *p* = 0.007) and the parietal region (*Z* = 2.15, *p* = 0.031). No significant differences were found in the frontal and temporal regions (all *p* > 0.05).

**Table 1:** rmANOVA results for EEG spectral power (μV^2^/Hz) across frequency bands under rhythmic and arrhythmic flicker conditions.

Frequency band	Rhythmic flicker (Mean ±SD)				Arrhythmic flicker (Mean ± SD)			
	Occipital	Parietal	Temporal	Frontal	Occipital	Parietal	Temporal	Frontal
Alpha	3.96 ± 4.04**	1.72 ± 1.68*	0.94 ± 0.69	3.30 ± 3.13	3.45 ± 3.74	1.70 ± 1.72	0.93 ± 0.71	3.17 ± 2.93
Beta	0.55 ± 0.260	0.22 ± 0.13	0.31 ± 0.20	1.24 ± 1.34	0.52 ± 0.24	0.21 ± 0.13	0.30 ± 0.19	1.23 ± 1.31
Delta	7.09 ± 8.85	3.98 ± 4.88	5.04 ± 6.79	17.07 ± 24.44	7.13 ± 9.46	3.93 ± 5.13	5.00 ± 6.97	15.89 ± 21.29
Theta	2.12 ± 1.37	0.97 ± 0.60	1.06 ± 0.74	5.99 ± 10.13	2.12 ± 1.27	0.97 ± 0.60	1.07 ± 0.78	6.25 ± 11.28

M ± SD, *p < 0.05, **p < 0.01.

Alpha power: rmANOVA revealed a significant main effect of experimental Condition (*F*_(1, 42)_ = 6.90, *p* = 0.012, η^2^_p_ = 0.14), with the overall alpha power being significantly higher under the rhythmic flicker condition (Condition 1, M = 2.35 μV^2^) than under the arrhythmic flicker condition (Condition 2, M = 2.19 μV^2^). The significant main effect of Region was also observed (*F*_(3, 126)_ = 19.630, *p* < 0.001, partial η^2^ = 0.30). Bonferroni-corrected post-hoc pairwise comparisons indicated significant differences in alpha power between most brain region pairs (all *p* < 0.05), with the exception of the frontal and parietal regions (*p* = 1.000). Additionally, a significant main effect of Electrode location was found (*F*_(2, 84)_ = 14.19, *p* < 0.001), reflecting systematic differences in alpha power across different electrodes. Moreover, two significant interaction effects were identified. First, a significant Condition × Region interaction (*F*_(3, 126)_ = 7.83, *p* < 0.001, η^2^_p_ = 0.15). Simple effects analysis was performed based on estimated marginal means. Multiple comparisons were corrected using the Bonferroni method unless otherwise noted. The results revealed that, under the rhythmic flicker condition, alpha power in the occipital region was significantly higher than the arrhythmic flicker condition, with a mean difference of 0.51 μV^2^ (*p* = 0.007, uncorrected). As the data from the parietal region violated the normality assumption, we further employed the Wilcoxon signed-rank test for analysis. The results showed a moderate enhancement in the parietal region (*Z* = 2.15, *p* = 0.031, uncorrected). No significant condition-related differences were observed in the frontal and temporal regions (all *p* > 0.05, Bonferroni-corrected). Second, a significant Region × Electrode interaction was observed (*F*_(6, 252)_ = 12.83, *p* < 0.001, η^2^_p_ = 0.21), indicating that the distribution pattern of alpha power across electrodes varied depending on the brain region.

**Figure 4:**
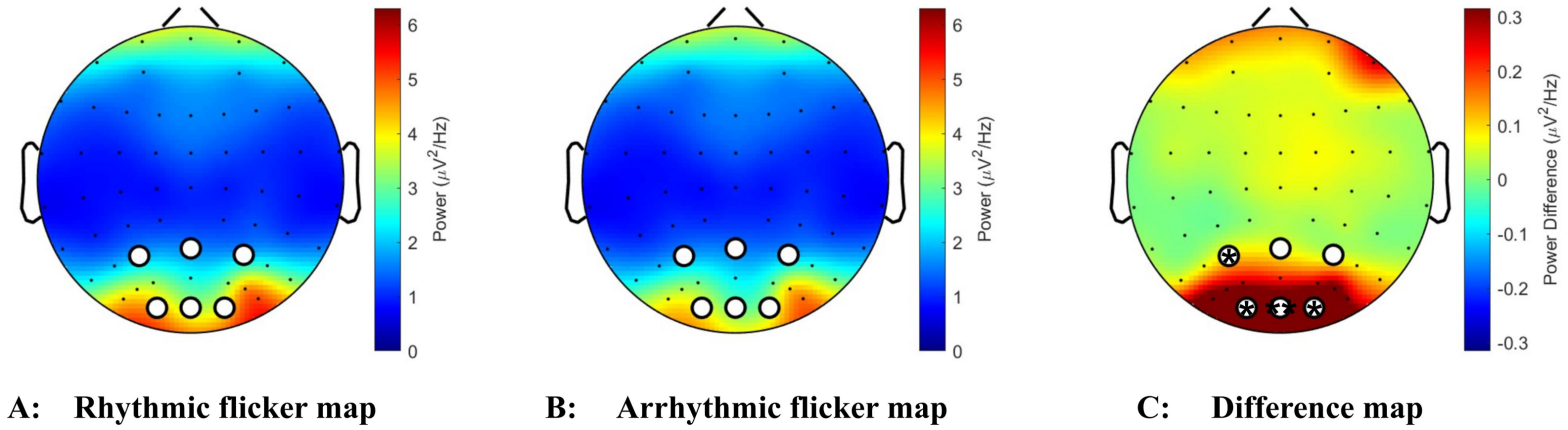
**(A–C)** Topographic distribution of alpha-band (8–13 Hz) power during visual flicker stimulation. **(A)** Power map under rhythmic flicker stimulation (predictable temporal pattern). **(B)** Power map under arrhythmic flicker stimulation (unpredictable temporal pattern). **(C)** Difference map (rhythmic – arrhythmic) showing regions of enhanced alpha activity during rhythmic stimulation. Color scales represent power (μV^2^/Hz) in A and B, and power difference (μV^2^/Hz) in panel **(C)**. Electrodes marked in white denote occipital sites (O1, O2, and Oz) and three parietal electrodes (P3, Pz, and P4). Significance markers indicate electrode locations where condition effects reached statistical significance (*p < 0.05, **p < 0.01). The occipital electrodes exhibit the most prominent condition effects.

Based on the significant Condition × Region interaction and Region × Electrode interaction in the alpha band, we conducted a whole-brain analysis to examine the differences between the two conditions across all brain regions. Given that the normality assumption was violated (Shapiro–Wilk test, *p* < 0.0001), we employed a Wilcoxon matched-pairs signed-rank test to compare alpha power between rhythmic and arrhythmic flicker conditions. The analysis revealed a highly significant difference (*Z* = 0.11, *p* = 0.0044, *n* = 43), indicating that alpha power was significantly higher in the rhythmic flicker condition (Figures 3A,B). Topographic analyses indicated that rhythmic versus arrhythmic visual flicker stimulation modulated both the spatial distribution and magnitude of alpha-band oscillatory activity in the brain, as shown in Figure 4. The topographic maps of alpha power (Figures 4A,B) revealed systematic differences in spatial patterns between the two conditions, with the difference map (Figure 4C) further demonstrating that rhythmic stimulation predominantly enhanced alpha activity in the occipital regions and the parietal region, particularly in the occipital area. This spatial specificity aligns with the Region main effect identified above, confirming that the occipital region exhibits the highest alpha power and the most pronounced condition-related modulation, followed by the parietal region.

Beta power: Analysis of neural activity in the beta frequency band revealed a significant main effect of Condition (*F*_(1, 42)_ = 4.83, *p* = 0.033, η^2^_p_ = 0.11). Beta power was significantly higher under the rhythmic flicker condition (Condition 1, *M* = 0.59 μV^2^) than under the arrhythmic flicker condition (Condition 2, *M* = 0.58 μV^2^), with a mean difference of 0.014 μV^2^. A significant main effect of Region was also found (*F*_(3, 126)_ = 26.06, *p* < 0.001, η^2^_p_ = 0.30). Bonferroni-corrected post-hoc pairwise comparisons indicated a descending regional distribution of beta power: frontal (Region 1, *M* = 1.28 μV^2^) > occipital (Region 4, *M* = 0.53 μV^2^) > temporal (Region 2, *M* = 0.31 μV^2^) > parietal (Region 3, *M* = 0.22 μV^2^). All inter-regional differences were statistically significant (all *p* < 0.001), except for the comparison between temporal and parietal regions (*p* = 0.008). Furthermore, a significant main effect of Electrode location was detected (*F*_(2, 84)_ = 23.80, *p* < 0.001), reflecting a progressive increase in beta power across the three electrode positions (Electrode 1, *M* = 0.49 μV^2^ < Electrode 2, *M* = 0.58 μV^2^ < Electrode 3, *M* = 0.69 μV^2^), with all pairwise comparisons reaching significance (all *p* ≤ 0.009). In addition, a significant Region × Electrode interaction was observed (*F*_(6, 252)_ = 7.76, *p* < 0.001), characterized by markedly higher beta power across all three electrodes within the frontal region (Electrode 1: 1.02 μV^2^; Electrode 2: 1.30 μV^2^; Electrode 3: 1.53 μV^2^) relative to other cortical regions, which exhibited lower beta power and reduced inter-electrode variations. By contrast, the Condition × Region (*F*_(3, 126)_ = 2.14, *p* = 0.099), Condition × Electrode (*F*_(2, 84)_ = 0.21, *p* = 0.813), and three-way Condition × Region × Electrode (*F*_(6, 252)_ = 0.39, *p* = 0.886) interactions were not statistically significant. This pattern indicates that the enhancing effect of rhythmic flicker stimulation on beta power is global and does not exhibit region-specific modulation observed in the alpha band. Finally, a between-participants test confirmed that the overall mean beta power (0.59 μV^2^; 95% confidence interval [CI]: 0.46–0.71) was significantly greater than zero (*F*_(1, 42)_ = 90.02, *p* < 0.001). Although the main effect of Condition on beta power achieved statistical significance (*p* = 0.033), the absolute difference between conditions was minimal (0.014 μV^2^) and no significant interactions with spatial factors (brain region and electrode location) were observed. Thus, the effect of rhythmic flicker condition on the beta band was marginal.

Delta and theta power: For delta-band activity, the analysis revealed a non-significant main effect of Condition (*F*_(1, 42)_ = 0.00, *p* = 0.0985, η^2^_p_ = 0.00), indicating that rhythmic flicker did not modulate the overall delta power. By contrast, the main effect of Region was significant, albeit with a modest effect size (*F*_(3, 126)_ = 7.14, *p* < 0.001, η^2^_p_ = 0.145). The Condition × Region interaction was non-significant (*F*_(3, 126)_ = 0.008, *p* = 0.999, η^2^_p_ = 0.000). Theta-band analysis revealed no statistically significant difference between the rhythmic and arrhythmic flicker conditions, with the main effect of Condition being non-significant (*F*_(1, 42)_ = 0.85, *p* = 0.363, η^2^_p_ = 0.02). The Condition × Region interaction also failed to reach statistical significance (*F*_(3, 126)_ = 1.04, *p* = 0.375, η^2^_p_ = 0.02). However, a significant main effect of Region was identified (*F*_(3, 126)_ = 9.99, *p* < 0.001, partial η^2^ = 0.19), indicating a clear frontal dominance of theta power. The highest amplitude was recorded in the frontal region (6.12 μV^2^), followed by the temporal (2.12 μV^2^), occipital (1.07 μV^2^), and parietal (0.97 μV^2^) regions. Exploratory uncorrected analyses at the electrode level revealed no significant differences between the two conditions.

In summary, the analyses of the delta, and theta frequency bands revealed no significant main effects of the rhythmic flicker condition and no significant Condition × Region interactions. For the beta band, while the main effect of Condition was significant, the Condition × Region interaction was not significant. Therefore, we focused our research emphasis on the alpha frequency band. The significant regional patterns observed for each band reflected their inherently well-established topographical distributions. These consistent null findings across the non-alpha bands provide strong evidence for the frequency specificity of the neural entrainment effect, which was exclusively observed within the alpha band.

### 3.3 Correlation analysis result

**Figure 5:**
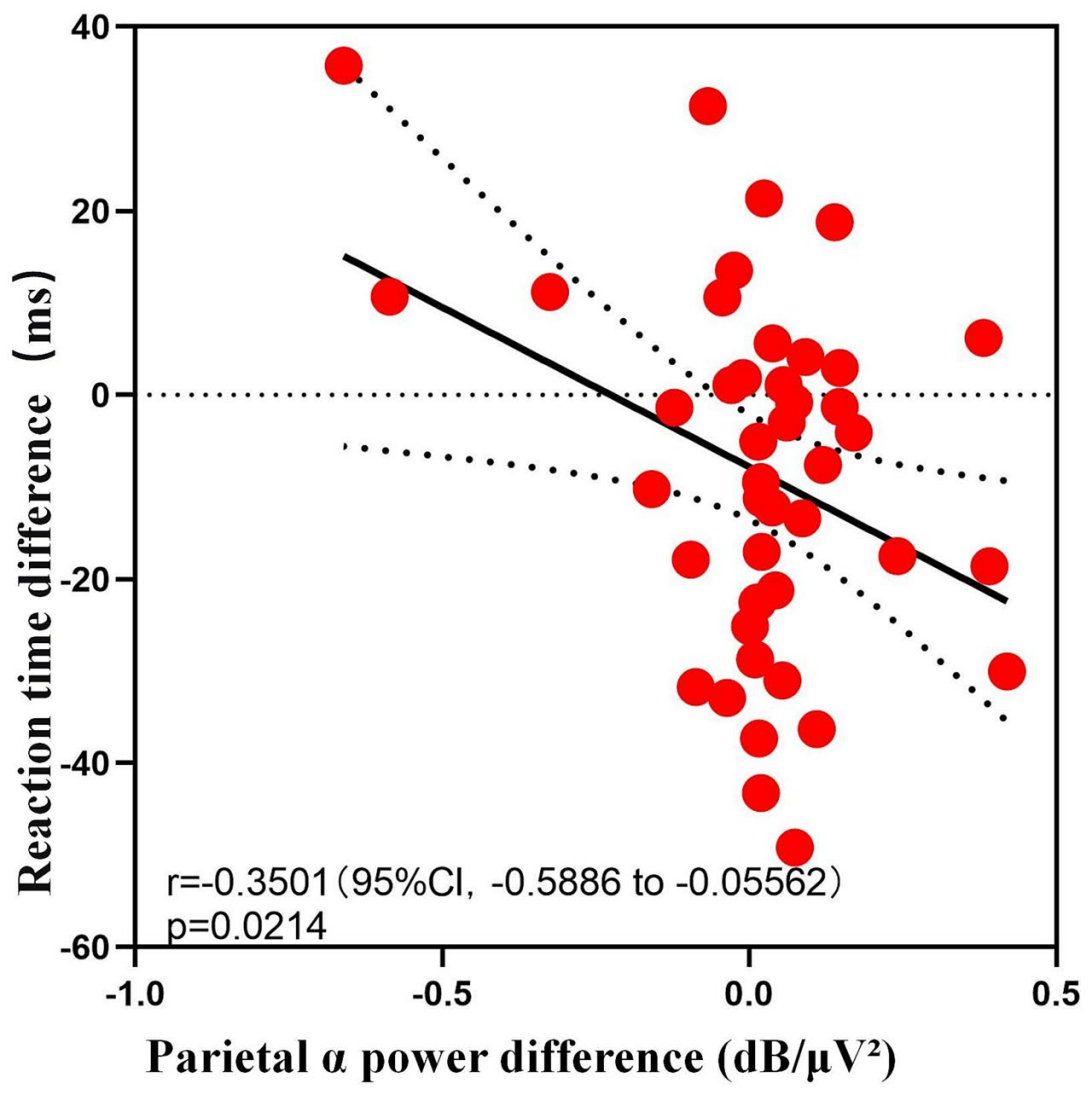
Scatter plot showing a significant negative correlation between parietal alpha power difference and reaction time difference.

Correlation analyses revealed a significant negative correlation between condition-related change in reaction time and the corresponding change in parietal alpha PSD (Pearson’s *r* = −0.35, *p* = 0.021; Spearman’s *ρ* = −0.347, *p* = 0.023), as shown in Figure 5. Specifically, participants who exhibited enhanced alpha activity in the parietal region showed stable associations with improved reaction time, suggesting a potential specific role of this brain area in regulating task-processing speed. Furthermore, alpha-band PSD changes across brain regions were strongly and positively correlated with one another (all *r* > 0.48, *ρ* > 0.46, *p* < 0.001), indicating that alpha oscillations exhibit systematic, cross-regional coordination during cognitive regulation. In contrast, no significant correlations were found between occipital alpha power and reaction time (*r* = −0.164, *p* = 0.294), accuracy (*r* = −0.075, *p* = 0.633), or perceptual sensitivity (d’) (*r* = 0.006, *p* = 0.97), highlighting the region-specific nature of the relationship between alpha oscillations and task performance.

## 4 Discussion

The present study systematically investigated the cognition-enhancing effects of individualized alpha frequency–based rhythmic visual entrainment during low-contrast visual search tasks and elucidated the underlying neural mechanisms by integrating behavioral and neurophysiological evidence. Behaviorally, individualized alpha entrainment produced concurrent improvements in reaction speed, task accuracy, and d’, indicating a genuine enhancement of visual search performance rather than a speed–accuracy trade-off. At the neural level, rhythmic visual entrainment selectively modulated alpha-band oscillatory activity, which were reflected by overall oscillatory power changes and a significant Condition × Region interaction spatially confined to the parietal cortex. This spatial specificity constitutes a central finding of the present study, as it directly corroborates and extends existing frameworks regarding the functional role of alpha oscillations. Specifically, Zhigalov and Jensen (2020) used source localization to demonstrate that, in attentional tasks, the functional “gating” or inhibitory role implemented by alpha oscillations is primarily supported by higher-order association cortices such as the parieto-occipital region rather than by primary visual cortex. Consequently, the parietal-specific alpha modulation observed in the present study strongly suggests that the cognitive enhancement induced by individualized alpha entrainment does not result from an amplification of primary visual input (sensory gain) but rather from the optimization of attentional selection and inhibitory control networks in downstream higher-order cortices—most prominently the parietal lobe. This marked spatial specificity, restricted to parietal regions, together with strict frequency specificity—manifested by the absence of “spillover” effects into functionally distinct bands such as beta or theta—constitutes a dual-selectivity chain of evidence. This indicates that improvements in behavioral performance originate from precise resonance and targeted modulation between the external rhythmic drive and a specific endogenous neural circuit, namely, the parietal-alpha loop. In summary, the present findings provide novel experimental support for the “frequency-matching” principle in complex cognitive tasks and underscore the potential of oscillation-targeted, non-invasive neuromodulation strategies for enhancing cognitive function.

The observed modulation of alpha oscillations aligns with the well-established principle that external rhythmic stimulation exerts frequency-dependent effects on neural activity (Li et al., 2019). Stimuli that closely match an individual’s endogenous oscillatory frequency—specifically, the IAF—are particularly effective in eliciting neural entrainment (Michael et al., 2023). The IAF measured at rest is relatively stable, showing moderate-to-excellent stability over intervals ranging from weeks to months (Kondacs and Szabó, 1999; Grandy et al., 2013a; Haegens et al., 2014). Previous research has indicated that maximal synchronization can be achieved when external rhythms match the frequency of intrinsic neural oscillations (Herrmann, 2001; Notbohm et al., 2016). In contrast, both behavioral effects and neural responses are significantly attenuated when the stimulation frequency deviates from the IAF by 1–2 Hz (Spaak et al., 2014; Samaha and Postle, 2015). The robust modulation effects observed in the present study likely arise from such precise frequency matching, highlighting the importance of individualized parameters in rhythm-based neuromodulation. Indeed, previous studies have demonstrated that matching visual flicker to the IAF substantially enhances perceptual learning compared with frequency-mismatched stimulation (Buzsáki and Draguhn, 2004). In the present study, systematic examination across multiple frequency bands provided clear evidence for the frequency specificity of alpha entrainment. Although beta-band activity showed significant overall power changes, these changes lacked spatial specificity and did not demonstrate a stable relationship with behavioral improvement. By contrast, alpha-band modulation was characterized by both a significant condition effect and precise regional specificity: significant pre–post differences were observed exclusively in the parietal region, and changes in parietal alpha power were negatively correlated with improvements in individual reaction speeds. This dual specificity—across both frequency and spatial dimensions— indicates that behavioral benefits are closely linked to the modulation of a specific frequency band (alpha) within a specific brain region (parietal cortex), without spillover into other functionally distinct frequency bands. Such a pattern strongly supports the “entrainment–resonance” model (Mathewson et al., 2009), according to which cognitive enhancement induced by rhythmic stimulation arises from selective resonance with intrinsic frequencies of specific circuits, rather than non-specific modulation (Helfrich et al., 2014; Michael et al., 2023), This frequency-specific mechanism is particularly relevant under challenging visual conditions, where noise filtering is critical for performance (Jensen and Mazaheri, 2010). In the parieto-occipital region, alpha oscillations are thought to implement a periodic inhibitory mechanism that facilitates such filtering by suppressing task-irrelevant information. Notably, the parietal cortex—where this alpha modulation was selectively observed in the present study—acts as a central hub for multiple large-scale brain networks. Therefore, modulation of parietal alpha activity may not only optimize local inhibitory gating but also influence the dynamic balance between systems such as the dorsal attention network and the default mode network (DMN).

The divergent response patterns across frequency bands observed in the present study are consistent with the current understanding of the functional specificity of brain oscillations. Alpha oscillations (8–13 Hz) are commonly linked to rhythmic inhibition in the visual cortex, attentional filtering, and perceptual temporal segmentation (Bonnefond and Jensen, 2025; Samaha and Postle, 2015; Karvat et al., 2024). Conversely, beta oscillations (13–30 Hz) are more closely associated with sensorimotor integration, cognitive maintenance, and long-range communication (Zaepffel et al., 2013), while theta oscillations (4–8 Hz) are primarily implicated in memory encoding and spatial navigation (Rozengurt et al., 2023; Tort et al., 2013). The significant condition main effect and a Condition × Region interaction in the alpha band observed in this study highlight the relative stability and functional independence of beta- and theta-band activities. Notably, although beta oscillation power was modulated at the global level (significant condition main effect), its spatial distribution pattern—reflected in the significant main effects of Region and Electrode and their interaction—remained stable. This suggests that beta activity may represent a more stable, trait-like neural baseline that is less susceptible to alterations caused by brief visual rhythmic entrainment.

Recent advances have provided deeper mechanistic insights into this frequency and spatial specificity. A 2025 study published in *eNeuro* reported that alpha-frequency transcranial alternating current stimulation (*α*-tACS) selectively enhanced dynamic coupling between alpha oscillation power and functional connectivity within the DMN, with no comparable effects in other frequency bands or networks (Ma et al., 2025). α-tACS may enhance α-synchronization within the DMN, thereby facilitating the generation of spontaneous associations and remote connections (improving fluency and flexibility) (Zhou et al., 2025). The DMN is a large-scale brain network associated with introspection and episodic memory retrieval (Varela et al., 2001; Buzsáki and Draguhn, 2004; Shanahan, 2010; Buckner and DiNicola, 2019). Given the region-specific alpha modulation observed in the present study—particularly involving the parietal cortex—future research should examine whether personalized visual alpha entrainment improves cognitive performance by strengthening coordination within specific large-scale networks, such as the interaction between the dorsal attention network (involved in visual processing) and the DMN.

From a neuroanatomical perspective, alpha rhythms are thought to originate primarily from corticothalamic circuitry, specifically through interactions between the thalamic reticular nucleus and cortical layer IV neurons—a circuit endowed with unique biophysical and resonance properties (Başar, 2012). By contrast, beta and gamma oscillations are more strongly dependent more on locally organized cortical inhibitory interneuron networks (e.g., basket cells) (Lamsa and Taira, 2003; Moca et al., 2014). Within this framework, our findings indicate that visual flicker stimulation preferentially resonates with and drives the specific corticothalamic circuit that is sensitive to visual input and responsible for alpha rhythm generation while exerting limited influence on locally generated beta or theta microcircuits.

Functionally, alpha oscillations are hypothesized to create discrete “temporal windows” for perception via periodic inhibitory phases that rhythmically gate cortical excitability (Jia et al., 2022; Jiang et al., 2023). Personalized alpha-frequency entrainment may induce phase resetting and synchronization of excitatory phases across large neuronal populations, thereby establishing periodic, low-noise, and high-excitability windows in task-relevant brain regions (Zuo and Wang, 2024). Such optimization likely enhances the signal-to-noise ratio of visual information at specific time points while minimally affecting oscillatory rhythms that subserve information processing at other temporal scales (e.g., slower memory integration linked to the theta band) or different functions (e.g., motor preparation associated with the beta band).

Correlational analyses further elucidated the nuanced associations between neural dynamics and behavioral enhancement. Specifically, condition-related differences in reaction time demonstrated a significant negative correlation with changes in parietal alpha-band (8–13 Hz) PSD, indicating a direct relationship between enhanced parietal alpha activity and improved processing speed. Concurrently, alpha-band PSD differences across brain regions exhibited systematically high positive intercorrelations, reflecting the cross-regional synergistic modulation of the alpha rhythm. These findings deepen our understanding of alpha oscillation mechanisms at multiple levels. First, they support the “periodic inhibition” hypothesis, suggesting that individualized alpha entrainment may optimize excitatory cycles in key brain regions via phase resetting, thereby improving the signal-to-noise ratio and efficiency of information-processing (Klimesch et al., 2007). Second, the tight coupling between the magnitude of behavioral improvement and neural changes in specific regions underscores the functional selectivity of the intervention. Coordinated whole-brain changes in alpha oscillations indicate a systemic regulatory process within large-scale networks, implying that the cognitive enhancement mechanism of individualized alpha entrainment embodies the dual characteristics of precise local modulation and global synergistic integration. In other words, it simultaneously optimizes local information processing through targeted alpha modulation in task-relevant regions and maintains systemic functional coherence by coordinating large-scale network activities. This insight also informs future research directions: given the established roles of alpha oscillations in large-scale network coordination, the observed neurobehavioral coupling and cross-regional synergy may reflect a more complex, multi-scale network regulatory mechanism. Future studies should employ brain network connectivity analyses (e.g., Granger causality and dynamic functional connectivity) to examine whether such coupling is mediated by changes in cross-regional functional connectivity or network-level interactions, thereby further clarifying the pathways of rhythmic neuromodulation at the system level.

Despite providing robust support for the cognitive enhancement effects of individualized alpha entrainment through a multi-level analytical approach, this study has several limitations. First, the analyses predominantly relied on frequency-domain (spectral) power measures; future work should incorporate phase-based analyses to uncover finer-grained neurodynamic mechanisms. Second, the current task design does not fully dissociate entrainment effects on distinct cognitive subprocesses, and the inclusion of more rigorous active control conditions will be necessary to definitively establish frequency specificity. Third, inferences regarding network-level mechanisms remain speculative and will require direct validation using connectivity-based analyses. Fourth, the present study recruited exclusively male participants, which limits the generalizability of our findings to the female population. In future research, it is necessary to recruit female participants according to gender balanced design to comprehensively evaluate the generalizability of visual entrainment effects. Finally, the homogeneous sample and laboratory-based task limit the generalizability and ecological validity of the findings. Future studies should examine these effects of diverse populations and real-world settings. Together, these considerations provide a clear roadmap for advancing the understanding and potential of rhythmic neuromodulation.

In summary, through systematic behavioral and neurophysiological experiments, this study demonstrated that visual rhythmic entrainment based on individualized alpha frequencies effectively enhanced low-contrast visual search performance. This enhancement is supported by a convergent chain of evidence—encompassing multidimensional behavioral improvements in reaction time, accuracy, and d’—and accompanied by selective modulation of alpha-band oscillatory activity. Crucially, rhythmic entrainment induced region-specific changes in alpha power without spillover into other frequency bands (e.g., beta, delta, or theta), underscoring its strict frequency specificity. Most notably, the significant negative correlation between reaction time differences and parietal alpha-band (8–13 Hz) PSD establishes a direct link between neural modulation and behavioral gain. Collectively, these findings illustrate that individualized alpha entrainment optimizes visual cognition–related cortical network states through dual frequency and spatial specificity, thereby enhancing the quality of information processing at the foundational level of perceptual encoding.

## Data Availability

The raw data supporting the conclusions of this article will be made available by the authors, without undue reservation.
