# Supplementary figures and images for: Rhythmic visual stimulation enhances visual search via occipito-parietal alpha modulation: an electroencephalographic study
Source: Front Neurosci. 2026 Apr 15;20:1780980. doi: 10.3389/fnins.2026.1780980 (PMC13125035; doi:10.3389/fnins.2026.1780980)

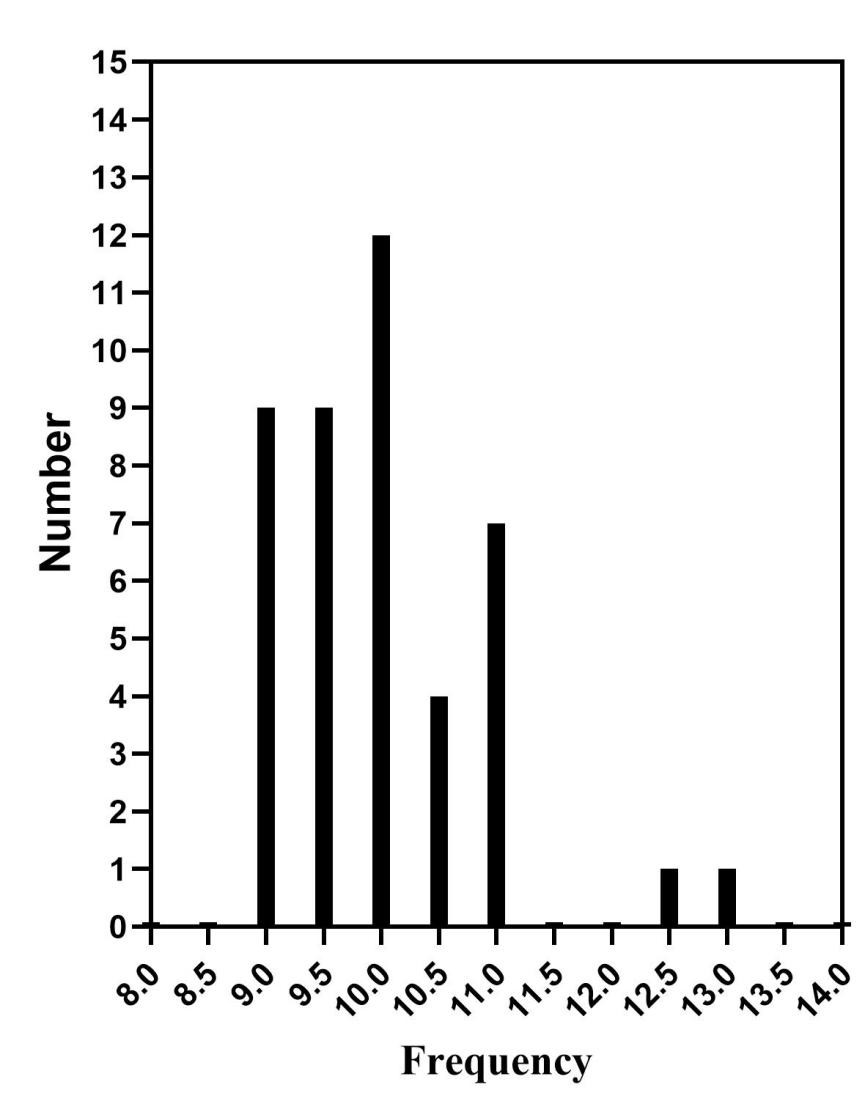

Supplement: Supplementary file 1 [file Image_1.jpeg]
