# Supplementary material for: Rhythmic visual stimulation enhances visual search via occipito-parietal alpha modulation: an electroencephalographic study
Source: Front Neurosci. 2026 Apr 15;20:1780980. doi: 10.3389/fnins.2026.1780980 (PMC13125035; doi:10.3389/fnins.2026.1780980)
Supplement: Supplementary file 4 [file Supplementary_file_1.docx]

Supplementary Material:

To further validate the robustness of IAF estimation, the present study analyzed the complete 180-s artifact-free EEG recording as a single continuous segment (original sampling rate: 1000 Hz, downsampled to 500 Hz after preprocessing). The power spectrum was computed from the averaged signal across five occipital electrodes (PO3, PO4, Oz, O1, O2), and the peak frequency within the 8–13 Hz range was identified and discretized to the nearest 0.5 Hz, consistent with the main analysis. As an independent validation, the FOOOF toolbox was employed for spectral parameterization of resting-state EEG, and the peak frequency from FOOOF was compared to the results obtained from the primary method(Donoghue et al., 2020). The IAF distribution of the 43 participants ranged from 9 to 13 Hz, with a mean of approximately 10.02 Hz, consistent with previous reports of adult IAF distributions. Notable inter-individual variability in IAF was observed, supporting the necessity of using individualized stimulation frequencies. Had a uniform 10 Hz flicker been applied to all participants, deviations of 1–3 Hz between the external stimulation frequency and the intrinsic alpha frequency would have occurred for individuals with lower (e.g., 9 Hz) or higher (e.g., 13 Hz) IAF, potentially compromising frequency alignment and entrainment efficacy.

The Gaussian smoothing method yielded a mean IAF of 9.98 ± 0.88 Hz (range: 8.26–12.53 Hz). Following discretization to 0.5 Hz steps, the actual stimulation frequencies applied in the experiment were distributed as follows: 9 Hz (n = 9), 9.5 Hz (n = 9), 10 Hz (n = 12), 10.5 Hz (n = 4), 11 Hz (n = 7), 12.5 Hz (n = 1), and 13 Hz (n = 1), with a mean of 10.02 ± 0.91 Hz (see Figure S1 for details). The FOOOF parameterization method produced a highly comparable mean IAF of 10.04 ± 0.89 Hz (range: 8.40–12.53 Hz). Participant-wise comparison revealed strong agreement between the two methods (Pearson's r = 0.975, p < 0.001). As shown in Figure S2(A), the paired line plot of IAF for 43 participants reveals nearly complete overlap between the two methods: orange solid lines represent the Gaussian smoothing method (original) and blue dashed lines represent the FOOOF method (validation), with light shading indicating minor differences. The difference distribution (Figure S2(B)) is overlaid with a fitted normal curve, showing an approximately normal distribution centered on zero.

To clearly illustrate the differences in power spectral density between the occipital and parietal regions, we generated Supplementary Figure S3, which specifically displays the alpha power spectral density differences in the occipital (O1, O2, Oz) and parietal (P3, P4, Pz) regions for each participant across the two stimulation conditions. Supplementary Figure S3 presents the paired individual data points for rhythmic versus arrhythmic conditions. Each dot represents one participant, with connecting lines indicating paired comparisons within subjects. The results reveal that the majority of participants exhibited elevated alpha power during rhythmic stimulation compared to arrhythmic stimulation in both the occipital and parietal regions. This pattern is consistent with the group-level findings reported in Table 1 and supports the robustness of the rhythmic stimulation effect across individuals.

**Figure S Legends**

**Figure S1.** Distribution of individual alpha peak frequency (IAF) across all 43 participants.

**Figure S2.** Validation of individual alpha peak frequency (IAF) estimated by Gaussian smoothing versus the FOOOF toolbox. (A): Participant-wise IAF comparison: orange solid lines (Gaussian smoothing method) and blue dashed lines (FOOOF method) show substantial overlap, with light shading indicating between-method differences; (B): IAF difference distribution: approximately normal distribution centered on zero, overlaid with a fitted normal curve, N = 43.

**Figure S3.** Individual alpha power spectral density in the occipital and parietal regions under rhythmic and arrhythmic conditions. Alpha power spectral density was obtained from occipital (Figure S(A): O1, O2, Oz) and parietal (Figure S(B): P3, P4, Pz) electrodes across the two stimulation conditions. Each dot represents one participant, connected by lines for paired comparison. The results show that rhythmic stimulation induced elevated alpha power in most participants.

**Reference**

DONOGHUE, T., HALLER, M., PETERSON, E. J., VARMA, P., SEBASTIAN, P., GAO, R., NOTO, T., LARA, A. H., WALLIS, J. D., KNIGHT, R. T., SHESTYUK, A. & VOYTEK, B. 2020. Parameterizing neural power spectra into periodic and aperiodic components. *Nat Neurosci,* 23**,** 1655-1665.
